# Two new species of *Caloptilia* (Lepidoptera, Gracillariidae) from New Caledonia inducing galls on *Glochidion billardierei* (Phyllanthaceae) and redescription of *C. xanthopharella* (Meyrick, 1880)

**DOI:** 10.3897/zookeys.1268.173885

**Published:** 2026-02-04

**Authors:** Antoine Guiguet, Erik J. van Nieukerken, David Giron, Barbara Gravendeel, Carlos Lopez-Vaamonde, Issei Ohshima

**Affiliations:** 1 Naturalis Biodiversity Center, PO Box 9517, 2300 RA Leiden, The Netherlands Laboratory of Insect Evolution and Diversity, Department of Life and Environmental Sciences, Kyoto Prefectural University Kyoto Japan; 2 Institut de Recherche sur la Biologie de l’Insecte, CNRS UMR 7261, Université François-Rabelais de Tours, UFR Sciences et Techniques, Tours, France Center for Frontier Natural History, Kyoto Prefectural University Kyoto Japan; 3 Institut de Systématique, Évolution, Biodiversité (ISYEB), UMR7205 Muséum national d’Histoire naturelle, CNRS, Sorbonne Université, EPHE, Université des Antilles, Paris, France Natural History Museum Vienna Vienna Austria; 4 Hortus botanicus Leiden, Leiden University, PO Box 9500, NL-2300 RA, Leiden, Netherlands Hortus botanicus Leiden, Leiden University Leiden Netherlands; 5 INRAE, UR633, Zoologie Forestière, F-45075 Orléans, France Naturalis Biodiversity Center Leiden Netherlands; 6 Natural History Museum Vienna, 2nd Zoological Department, Burgring 7, 1010 Vienna, Austria Institut de Systématique, Évolution, Biodiversité (ISYEB), UMR7205 Muséum national d’Histoire naturelle, CNRS, Sorbonne Université, EPHE, Université des Antilles Paris France; 7 Laboratory of Insect Evolution and Diversity, Department of Life and Environmental Sciences, Kyoto Prefectural University, Sakyo, Kyoto Institut de Recherche sur la Biologie de l’Insecte, CNRS UMR 7261, Université François-Rabelais de Tours, UFR Sciences et Techniques Tours France; 8 Center for Frontier Natural History, Kyoto Prefectural University, Sakyo, Kyoto, 606–8522 Japan INRAE Orléans France; 9 Kyoto Botanical Garden, Sakyo, Kyoto, 606–0823 Japan Kyoto Botanical Garden Kyoto Japan

**Keywords:** Australia, DNA barcoding, endemism, frass disposal, host plant use, sympatry, taxonomy

## Abstract

New Caledonia is a biodiversity hotspot with high levels of micro-endemism, yet its gracillariid fauna remains poorly documented. Here, two new species of *Caloptilia* Hübner, 1825 (Gracillariidae) are described from *Glochidion* J.R.Forst. & G.Forst. (Phyllanthaceae) host plants in Parc des Grandes Fougères, New Caledonia: *Caloptilia augeas* Guiguet, Lopez-Vaamonde, van Nieukerken & Ohshima, **sp. nov**., and *Caloptilia ceryneia* Guiguet, Lopez-Vaamonde, van Nieukerken & Ohshima, **sp. nov**. Both species induce leaf galls on *Glochidion billardierei* Baill., co-occurring on the same host species, sometimes even on the same leaf. They exhibit distinct wing patterns, but very similar male and female genitalia, and DNA barcoding supports their status as separate species. These findings provide evidence for potential within-host sympatric speciation, as documented in other gall-inducing insects. The larval biology of *C. augeas* and *C. ceryneia* reveals a unique frass disposal behaviour, whereby waste is excreted through a hole and the aperture is subsequently sealed—an adaptation not previously reported in gall-inducing Lepidoptera. Our findings double the known number of gall-inducing species in Gracillariidae, highlighting that this life history strategy may be more common than currently appreciated. We also provide new information on distribution and host plants of *Caloptilia xanthopharella* (Meyrick, 1880), a leaf roller found on the same host plant, *G. billardierei*. These findings mark the first records of the subfamily Gracillariinae in New Caledonia. This study underscores the underexplored diversity of New Caledonian gracillariids and emphasises the conservation value of Parc des Grandes Fougères. Further surveys in the Indo-Pacific region may reveal additional yet undescribed *Caloptilia* species associated with Phyllanthaceae and help clarify the evolutionary mechanisms underpinning their diversification.

## Introduction

With 325 species, *Caloptilia* Hübner, 1825 is the second largest genus in the family Gracillariidae Stainton, 1854, with the majority of species recorded from the Holarctic region (De Prins and De Prins 2024). Within this genus, species show a high degree of host specificity, often being associated with a single host plant species or genus (Lopez-Vaamonde et al. 2021). However, *Caloptilia* as a whole demonstrates remarkable ecological versatility, with species feeding on over 15 host plant families, suggesting that host shifts have occurred frequently throughout its evolutionary history (Nakadai and Kawakita 2016; Li et al. 2022).

The larval biology of *Caloptilia* is typically characterised by a sequential lifestyle involving leaf-mining and sap-feeding in the early instars, followed by various forms of leaf manipulation, collectively referred to as “leaf-rolling.” This behaviour takes several distinct forms: rolling cut portions of leaves (roller-type 1), folding or stacking entire leaves (roller-type 2), and curling the leaf tip or lobes (roller-type 3) (Li et al. 2022). However, three known species deviate from this pattern by inducing galls, a rare strategy within Gracillariidae (Guiguet et al. 2019). The first gall-inducing species is the Nearctic *Kallia murtfeldtella* (Busck, 1904), a stem borer that forms galls on *Penstemon* (Plantaginaceae). On the basis of its isolated position in the phylogeny (Kawahara et al. 2017; Li et al. 2022), and its bionomy, it was recently transferred from *Caloptilia* to *Kallia* De Prins, Sruoga & Zwick, 2025. The second is the recently described Australian species *Kallia myopora* De Prins, Sruoga & Zwick, 2025, whose larvae tunnel in the stem of *Myoporum insulare* (Scrophulariaceae) making a gall (De Prins et al. 2025). The third species is *C. cecidophora* Kumata, 1966, associated with *Glochidion rubrum* Blume, *G. acuminatum* Müll. Arg., and *G. obovatum* Siebold & Zucc. (Phyllanthaceae) (Kumata 1966). In this species, the first two larval instars are leaf-miners, but gall induction begins during the third instar with the formation of a tentiform mine which stimulates the proliferation of plant tissue (Guiguet et al. 2019). Notably, *C. cecidophora* has a sixth larval instar, a feature absent in most other *Caloptilia* species (Guiguet et al. 2019). Although initially classified in a separate subgenus, *Cecidoptilia* Kumata, 1982, due to its highly reduced larval chaetotaxy (Kumata 1982), molecular phylogenies suggest a closer relationship between *C. cecidophora* and other *Caloptilia* species feeding on *Glochidion*, suggesting a closer evolutionary relationship within this host-associated clade (Nakadai and Kawakita 2016).

Historical literature further suggests the presence of additional undescribed gall-inducing *Caloptilia* species. Reports from the Philippines, Malaysia, and Indonesia describe galls on *Glochidion* species resembling those induced by *C. cecidophora*, in areas where this species is not known to occur (Uichanco 1919; Houard 1922; Docters van Leeuwen-Reijnvaan and Docters van Leeuwen 1926). Given the broad distribution of the genus *Glochidion*, comprising approximately 350 species across mainland Asia, Australia, and Oceania (Bouman et al. 2022), the existence of additional, undescribed gall-inducing *Caloptilia* species throughout the Indo-Pacific region is plausible. One such candidate is *Caloptilia hercoscelis* (Meyrick, 1939) from Fiji, reportedly a gall-inducer on an unidentified host plant, but otherwise its lifestyle remains poorly documented (Meyrick 1939).

In this paper, we describe two new sympatric gall-inducing species of *Caloptilia* from New Caledonia that induce leaf galls on *Glochidion billardierei* Baill. In addition, we report new distributional and host plant data for a third species, *Caloptilia xanthopharella* (Meyrick, 1880), which makes leaf rolls on the same host plant. These are the first records of the subfamily Gracillariinae for New Caledonia.

Recent phylogenetic studies have revealed that *Caloptilia*, as currently circumscribed, is polyphyletic (Kawahara et al. 2017; Li et al. 2022). In these analyses, *C. cecidophora* forms a clade sister to a large clade that includes various other genera as well as *Caloptilia* s. str. However, no formal taxonomical changes have been proposed to resolve this polyphyly. A future reclassification may involve either expanding *Caloptilia* to include related genera such as *Sabulopteryx* Triberti, 1985, *Systoloneura* Vári, 1961, *Gracillaria* Haworth, 1828, *Povolnya* Kuznetzov, 1979, *Euspilapteryx* Stephens, 1835, *Eucalybites* Kumata, 1982, or alternatively, splitting the genus, potentially elevating *Cecidoptilia* from subgenus to genus to accommodate the *Phyllanthaceae*-associated species.

## Materials and methods

### Sampling

**Figures 1–3.**
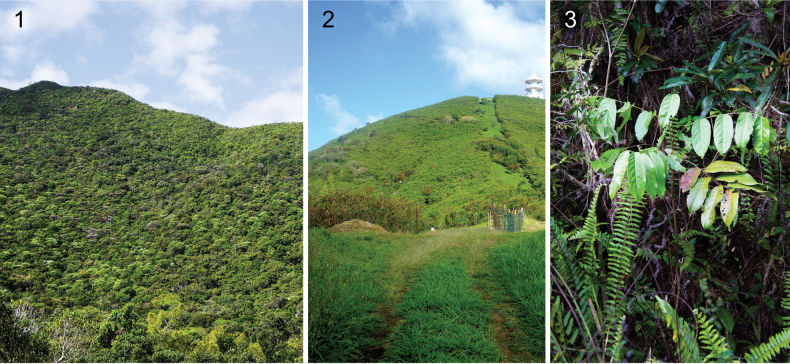
Habitat of *Caloptilia augeas* sp. nov., *C. ceryneia* sp. nov. and *C. xanthopharella* (Meyrick, 1880). **1**. The New Caledonian rain forest, Parc des Grandes Fougères; **2**. Disturbed forest in Noumea, near Parc Zoologique & Forestier; **3**. *Glochidion billardierei* Baill. (Phyllanthaceae), host plant of the three species.

Voucher adult specimens were obtained by rearing larvae (41 reared individuals) or directly collected by light trapping (three individuals) (Suppl. material 1). Larval sampling and light trapping were carried out in two areas of the Southern Province of New Caledonia: Parc des Grandes Fougères and at Nouméa (Figs 1, 2, Suppl. material 1) by AG, IO, and CLV. Larvae were collected together with the rolled or gall-induced leaves from their host plant, *Glochidion billardierei* Baill. (Fig. 3), and collected galls and leaf rolls were individually reared in a clear plastic case (135 × 85 mm) using the method described in Lopez-Vaamonde et al. (2021). *Glochidion billardierei* (Fig. 3) is a pioneer shrub or tree occurring in secondary rain forests and is endemic in New Caledonia, including the Loyalty islands (https://endemia.nc/flore/fiche8988).

### Morphology and nomenclature

The adults were photographed with a motorised Zeiss SteREO Discovery V12 or V20 and a AxioCam 305 colour camera, using Carl Zeiss ZEN 3.10 software. Genitalia were photographed with a Axio Imager.M2 or a manually operated Axioskop, with the same camera and software. Slide specimens of genitalia were prepared largely following Robinson (1976). After maceration in KOH and cleaning, Chlorazol Black E and Phenosafranin were used as stains and the genitalia were embedded in euparal on slide. Genitalia slides were prepared by AG and some by EvN, for practical reasons they all received an EvN slide number. This number corresponds to the RMNH registry number by adding 20,000 (the specimen with slide EvN5600 has registry number RMNH.INS.25600). Terms for genital morphology follow Klots (1970) and Kristensen (2003). The genital illustrations of *Caloptilia cecidophora* in Kumata (1982) were used for comparison. The scientific name of the host plant follows the World Flora Online website (https://www.worldfloraonline.org). Measurements of moths and genitalia were taken on microphotographs using the measurement extension in Carl Zeiss ZEN 3.10 software.

### Specimen depositories

**KPU** Kyoto Prefectural University, Kyoto, Japan;

**RMNH**Naturalis Biodiversity Center, Leiden, The Netherlands;

**MNHN** Muséum national d’Histoire naturelle, Paris, France;

**NHMUK** The Natural History Museum, London, UK;

**NHMW** Naturhistorisches Museum Wien, Austria.

### DNA barcoding

We removed a single hind leg from each of the 44 dried adult specimens. We placed the tissue samples in 96-well plates and shipped these to the Canadian Centre for DNA Barcoding (CCDB, Biodiversity Institute of Ontario, University of Guelph) for complete laboratory processing. DNA extraction was performed by a solid phase reversible immobilisation (SPRI) method. PCR-amplified samples were sequenced using single molecule real-time (SMRT) processing on a PacBio Sequel sequencing platform (Pacific Biosciences, Menlo Parc – California, United States of America) (Hebert et al. 2018).

Sequences were assigned automatically to Barcode Index Numbers (BIN’s) in the Barcode of Life Data Systems, v. 4 (BOLD) (Ratnasingham and Hebert 2013). Neighbor-joining (NJ) trees, used to visualise genetic distances within and between species and sequence clusters, were generated with BOLD’s workbench (Suppl. material 3). DNA sequences, along with the voucher data, images, and trace files, are stored in the BOLD database (Ratnasingham and Hebert 2007, https://www.barcodinglife.org) and the sequences were also deposited in GenBank (Suppl. material 1). In addition, we obtained 15 Australian sequences from BOLD belonging to *C. xanthopharella* (Suppl. material 1). All barcode records are publicly available in the BOLD data set “DS-CALNEO”, accessed at https://doi.org/10.5883/DS-CALNEO.

## Systematic account

### Gracillariinae Stainton, 1854

***Caloptilia* Hübner, 1825**

#### Caloptilia augeas

Taxon classificationAnimaliaLepidopteraGracillariidae

Guiguet, Lopez-Vaamonde, van Nieukerken & Ohshima, sp. nov.

BBCEF45A-D466-52C0-A36C-509BA6C3933F

https://zoobank.org/8CC15AC0-C40C-4B76-A832-20C3BB62F8AD

Figs 4–10, 21, 23, 24, 29–31, 38–42

##### Type material.

***Holotype***. New Caledonia • ♂; Province Sud, Farino, near La Fao, Parc des Grandes Fougères; 21.626°S, 165.759°E; alt. 430 m; 13 Sep. 2018; 23 Sep. 2018 em. [adult emergence]; Ohshima, Issei et al. leg.; Rearing No. IsO-1058; Host: “*Glochidion billarideri*” [recte billardierei]; DNA extraction No. IO-873; Genitalia slide: EvN5562; RMNH.INS.25562; RMNH. ***Paratypes*** (12 ♂ 15 ♀). New Caledonia, Same collecting data as holotype • 2 ♂; 26 & 20 Sep. 2018 em.; DNA extraction No.: IO-875, IO-871 & IO-966; Genitalia slides EvN5563, EvN5648; RMNH.INS.25563; RMNH.INS.25648 • 3 ♀; 22 & 29 Sep. 2018 em.; DNA extraction No.: IO-870, IO-881, IO-882 & IO-965; Genitalia slides EvN5561, EvN5560; RMNH.INS.1557477, RMNH.INS.25560, RMNH.INS.25561 • 2 ♂; 1 Oct. 2018 em.; DNA extraction No.: IO-886, IO-887; MNHN • 3 ♀; 18, 26 & 29 Sep. 2018 em.; DNA extraction No.: IO-872, IO-874, IO-880; MNHN • 1 ♂ 1 ♀; 28 Sep., 1 Oct 2018 em.; DNA extraction No.: IO-876, IO-888; NHMUK • 3 ♂; 1 & 11 Oct. 2018 em.; DNA extraction No.: IO-883, IO-885, IO-934; KPU • 5 ♀; 23 Sep. – 5 Oct. 2018 em.; DNA extraction No.: IO-867, IO-868, IO-877, IO-878, IO-933; KPU • 2 ♂; 1 & 3 Oct. 2018 em.; DNA extraction No.: IO-884, IO-932; NHMW •2 ♀; 22 & 29 Sep. 2018 em.; DNA extraction No.: IO-869, IO-879; NHMW •1 ♂ 1 ♀; Province Sud, Farino, Parc des Grandes Fougères, Camp de la Houe; 21.6109°S, 165.755°E; alt. 382 m ; 14 Sep. 2018; Ohshima, Issei et al. leg.; light trap; DNA extraction No.: IO-865 (♂), IO-866 (♀); MNHN • 1 ♂; Same data as previous; DNA extraction No.: IO-864; NHMUK.

##### Diagnosis.

**Figures 4–10.**
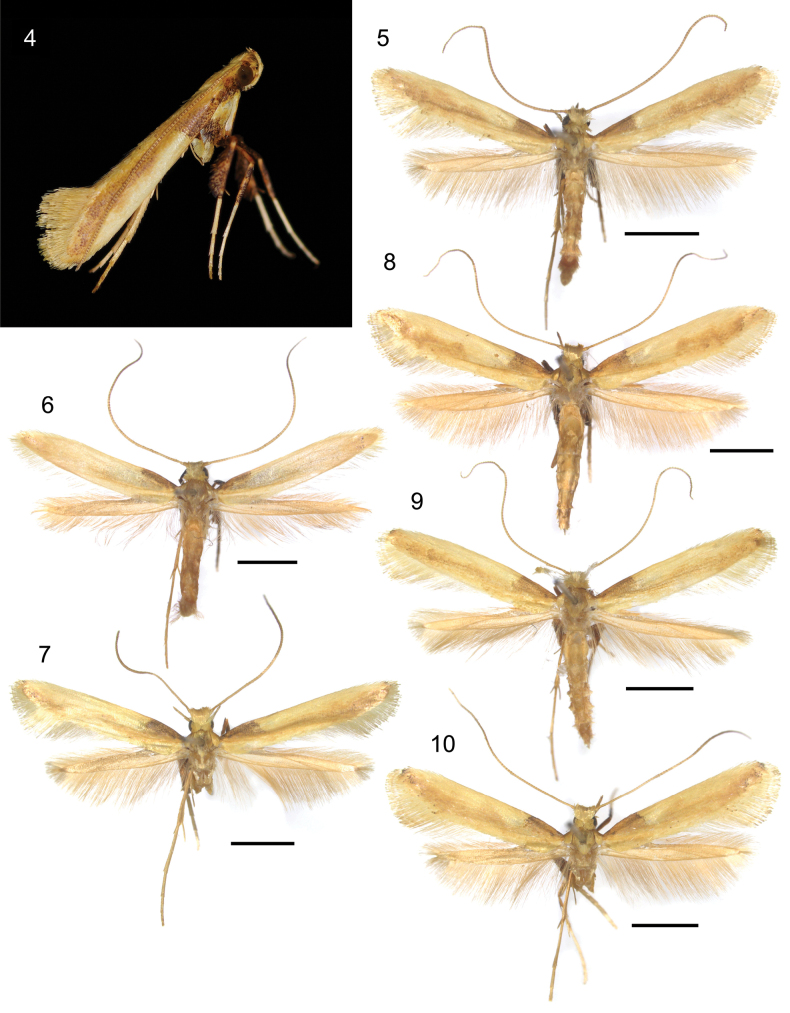
Habitus of adult *Caloptilia augeas* sp. nov. **4**. Resting posture; **5**. Paratype, male RMNH.INS.25563; **6**. Holotype, male, RMNH.INS.25562; **7**. Paratype, male, RMNH.INS.25648; **8**. Paratype, female, RMNH.INS.25561; **9**. Paratype, female, RMNH.INS.1557477; **10**. Paratype, female, RMNH.INS.25560. Scale bars: 2 mm.

*Caloptilia augeas* is distinguished from other *Caloptilia* species by the forewing with the ambiguously margined costal elongated large metallic gold patch and by the metallic gold hind wing (Figs 4–10). Despite the obvious difference in the wing pattern, both male and female genital morphology resembles *C. ceryneia* and *C. cecidophora*. However, *C. augeas* is distinguished from the former by the rounded ventral margin of valva and from the latter by the rounded terminal margin of valva in the male genitalia. In the female genitalia, *C. augeas* is clearly distinguished from the two species by large L-shaped signa. Larvae induce large leaf galls on *Glochidion billardierei*, and the galls protrude on the adaxial side with an opening on the abaxial side (Figs 38–42).

##### Description.

***Habitus*** (Figs 4–10). Forewing length 5.44–6.24 mm (5.44 mm in holotype, 5.84 mm on average of 6 specimens). No sexual dimorphism exhibited.

***Head***: Vertex metallic gold. Antenna 1.1× longer than forewing, yellow, annulated with brown. Labial palpus upcurved, metallic gold, brown apically.

***Thorax***: Tegula metallic gold. Forewing rather broad, with ground of light brown scales; basal 1/4 of costa ochreous-brown with a minute blackish spot near base; large elongated metallic gold patch ranging from 1/4 to apex along costa, narrowed at 1/2, and ambiguously margined by light brown scales except at the proximal margin where edged by dark brown scales; dorsum before tornus metallic gold, shading into light brown scales towards fold, and some scales with darkened tips around tornus to apex; fringe metallic gold, with three lines. Hindwing metallic gold, with greyish brown scales along costa from base to middle part; fringe metallic gold. Fore- and midlegs with coxa ochreous-brown; tarsi ochreous-white; hindleg with coxa metallic gold.

***Abdomen***: terga lustrous brass; sterna lustrous brass.

***Male genitalia*** (Figs 23, 24): Tegumen about as long as vinculum, poorly sclerotised. Valva elongate, upcurved, distally dilated, straight on terminal margin, setose on almost all the internal ventral surface of cucullus, naked distally. Vinculum a little more than 2/3 as long as valva. Phallus ~ 1.3 × as long as vinculum, needle-shaped, without cornuti; phallus length 595–645 µm.

***Female genitalia*** (Figs 29–31): Papilla analis with numerous spine-like setae on dorso-caudal corner besides the usual slender setae, posterior edge slightly convex more sclerotised. Antrum poorly developed. Ductus bursae long, slender, membranous. Ductus seminalis meets ductus bursae close to ostium. Corpus bursae elongate-ovoid in form, wrinkled; a pair of well-developed signa curved, sabre-shaped, protruding externally, as long as the diameter of the corpus bursae section. Signa length 235–320 µm.

##### Biology.

The species is known to feed exclusively on *Glochidion billardierei* Baill. (Phyllanthaceae). Larvae begin their development as sap feeding leaf miners (Fig. 39) creating an irregular gallery in the lower epidermis of the leaf. Subsequently, they induce the formation of prominent galls that protrude from the adaxial (upper) surface of the leaf (Fig. 38). Each gall has a small opening on the abaxial (lower) side, which the larva uses to expel frass from the larval chamber. Once waste removal is complete, the opening is sealed with a silk seal (Figs 40–42). Right before pupation, the larva digs the future adult exit hole below the adaxial surface of the gall on the extremity opposed to the frass hole, leaving only a circular membrane of epidermal cuticle. The larva pupates inside the gall. The adults emerged between 20 September and 11 October, suggesting that at least some of the collected galls contained pupae.

##### Distribution.

So far, the species is only known from two sites within the Parc des Grandes Fougères in the South Province of New Caledonia.

##### Etymology.

The specific name augeas refers to the fifth Labour of Hercules in the Greek mythology. The larval behaviour of frass cleaning in the gall reminds this Labour that consisted in cleaning the stables of king Augeas (Αὐγέας). This name comes from the word “αὐγή” that designates “sun shine” and “reflection of a shiny object” in Classic Greek (Bailly 1899), referring to the bright colour of the adult wings. The epithet is to be regarded as a noun in apposition.

##### DNA barcodes.

Twenty-eight barcodes of the same population form a single BIN BOLD:AEC4316, with an average distance of 0.2% and a maximum distance of 0.64%; there is a 3.69% (*p*-distance) difference to its nearest-neighbour BOLD:AEC4478 (*C. ceryneia*, Suppl. materials 1–3).

#### Caloptilia ceryneia

Taxon classificationAnimaliaLepidopteraGracillariidae

Guiguet, Lopez-Vaamonde, van Nieukerken & Ohshima, sp. nov.

DDA7D4D4-C453-5A7E-9A9B-D00CA378E094

https://zoobank.org/33FEA7C2-FE06-482B-BF37-299C2BA4D695

Figs 11–14, 22, 25, 26, 32–34, 43–45

##### Type material.

***Holotype***. New Caledonia • ♂; Province Sud, Farino, near La Fao, Parc des Grandes Fougères; 21.626°S, 165.759°E; alt. 430 m; 15 Sep. 2018; 17 Sep. 2018 em. [adult emergence]; Ohshima, Issei et al. leg.; Rearing No. IsO-1072; Host: “*Glochidion billarideri*” [recte billardierei] “(Same as IsO-1058)”; DNA extraction No.: IO-849 & IO-963; Genitalia slide: EvN5650; RMNH.INS.25650; RMNH. ***Paratypes*** (1 ♂ 1 ♀). New Caledonia • 1 ♂; same collecting data as holotype; 30 Sep. 2018 em.; DNA extraction No.: IO-850 & IO-964; Genitalia slide: EvN5564; RMNH.INS.25564; RMNH • 1 ♀; Province Sud, Nouméa, Parc Zoologigue & Forestier; 22.2582°S 166.458°S; alt. 83 m; 03 Mar. 2019; 14 Mar. 2019 em.; Issei Ohshima leg.; Rearing No. IsO-1089; Host: “*Glochidion billarideri*” [recte billardierei]); DNA extraction No.: IO-995; Genitalia slide: EvN5565; RMNH.INS.25565; RMNH.

##### Diagnosis.

**Figures 11–14.**
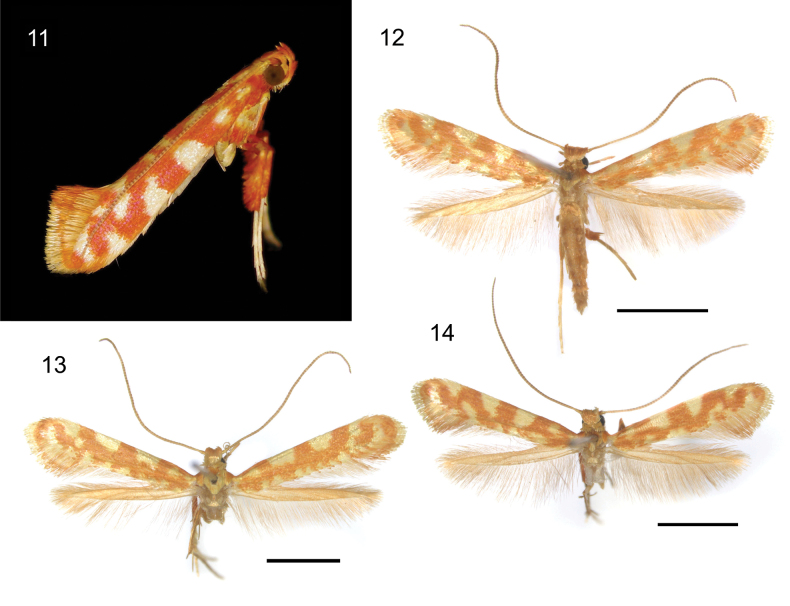
Habitus of adult *Caloptilia ceryneia* sp. nov. **11**. Resting posture, holotype male, RMNH.INS.25650; **12**. Paratype, female, RMNH.INS.25565; **13**. Paratype, male, RMNH.INS.25564; **14**. Holotype, male, RMNH.INS.25650. Scale bars: 2 mm.

The red-orange colour of the forewing of *C. ceryneia* is unique in *Caloptilia*, as well as the lustrous yellow blotch pattern (Figs 11–14). The male and female genitalia are very similar to those of *C. augeas*, but this species is clearly distinguished from *C. augeas* by the straight ventral margin of valva in the male genitalia and by the short-straight back-knife shaped signa in the female genitalia. Larvae induce small leaf galls on *Glochidion billardierei*, and the galls protrude equally on the adaxial and abaxial sides with an opening on the abaxial side (Figs 43–45).

##### Description of adult.

***Habitus*** (Figs 11–14). Forewing length 4.35–4.83 mm (4.83 in holotype). No sexual dimorphism exhibited.

***Head***: Vertex red-orange. Antenna 1.1 longer than forewing, yellow, annulated with brown. Labial palpus upcurved, each segment red-orange and basally yellow.

***Thorax***: Tegula anterior half red-orange shading into yellow laterally, posterior half lustrous yellow. Forewing rather broad, with ground of red-orange scales; few yellow scales at the base; a small lustrous yellow spot near the base, reaching dorsum but not costa; first costal blotch from basal 1/4 to 1/3, lustrous yellow, connected with a dorsal spot or unconnected (holotype); second costal blotch from beyond middle to 2/3, lustrous yellow, sometimes triangular shaped, not reaching dorsum; third costal blotch ~ 1/2 the size of second blotch, lustrous yellow; a series of lustrous yellow spots of variable shapes below the third costal blotch, sometimes merging with it; dorsum red-orange with 2–5 lustrous yellow spots of variable shapes; first line in fringe red-orange shading to lustrous yellow, second line in fringe lustrous yellow shading to red-orange posteriorly, third line in fringe lustrous yellow shading to red-orange. Hindwing lustrous bronze; fringe lustrous bronze. Fore- and midlegs with coxa red-orange; tarsi lustrous yellow; hindleg with coxa lustrous yellow.

***Abdomen***: terga lustrous bronze; sterna lustrous bronze.

***Male genitalia*** (Figs 25, 26): Tegumen slightly shorter than vinculum, poorly sclerotised. Valva elongate, upcurved, distally and dorsally dilated, straight on terminal margin, setose on almost all the internal ventral surface of cucullus, distally and apically naked. Vinculum a little less than 2/3 of as long as valva, gradually tapering toward obtusely pointed apex. Phallus ~ 1.2 × as long as vinculum, needle-shaped, without cornuti; phallus length 470–500 µm.

***Female genitalia*** (Figs 32–34): Papilla analis with a small number of spine-like setae on dorso-caudal corner besides usual slender setae, posterior edge straight with a concave region on half of its length. Ductus bursae long, slender, membranous. Ductus seminalis meets ductus bursae close to ostium. Corpus bursae piriform, wrinkled; a pair of signa curved, shaped like a short straight back knife, slightly protruding externally, with denticulation along the inner curve, shorter than the radius of the corpus bursae section. Signa length 90–92 µm.

##### Biology.

This species was found in the New Caledonian rain forest, where its host plant, *Glochidion billardierei* Baill. (Phyllanthaceae) grows. The larvae of this species induce small leaf galls that protrude equally on both the adaxial and abaxial side (Figs 43–45). Similar to *C. augeas*, the galls have a small opening on the abaxial side, which the larvae use to expell their frass out of the larval chamber. Once they have done this, they seal the opening with a silk membrane. Early instars are assumed make epidermal leaf-mines before inducing a gall. Right before pupation, the larva digs the future adult exit hole below the adaxial surface of the gall on the extremity opposed to the frass hole, leaving only a circular membrane of epiderm cuticle. The larva pupates inside the gall. The adults from the Parc des Grandes Fougères, galls collected on 15 September, emerged on 17 and 30 September; the one from Noumea, collected 3 March, emerged 14 March.

##### Distribution.

The species is currently known from only two localities: two individuals found in Parc des Grandes Fougères and a single individual was recorded at the Botanical Gardens in Nouméa.

##### Etymology.

The specific name ceryneia refers to the ancient Greek city of Ceryneia or Keryneia (Κερύνεια), the location of the Ceryneian Hind from Greek mythology, which features in the third Labour of Hercules. The moth’s striking yellow and red wing pattern evokes the golden antlers and bronze hooves of the mythical deer. Its cryptic gall-inducing behaviour also recalls the elusive nature of the Ceryneian hind, which was known for its ability to remain hidden. The epithet is to be treated as a noun in apposition.

##### DNA barcodes.

All three specimens were barcoded (IO-849, IO-850, IO-995), forming a single BIN, BOLD:AEC4478 (Suppl. materials 1, 2). Its nearest neighbour is BIN BOLD:AEC4316, formed by the 28 barcodes of *Caloptilia augeas* at 3.69% (*p*-distance) (Suppl. materials 2, 3).

##### Remarks.

Both female and male genital morphology of this species is similar to that of *C. cecidophora*, suggesting the close relationship among the three gall-inducing species. According to the original description, *C. hercoscelis* has greyish fore- and hindwings, indicating that the present two new species are clearly distinct species.

#### Caloptilia xanthopharella

Taxon classificationAnimaliaLepidopteraGracillariidae

(Meyrick, 1880)

929D3067-BBE0-5604-A250-3AB5A24EC79F

Figs 15–20, 27, 28, 35–37, 46, 47

*Grac*. [*ilaria*] *xanthopharella* Meyrick, 1880: 141. 3 syntypes [Australia, New South Wales], Sydney (NHMUK: BMNH(E)1324995, BMNH(E)1411610, BMNH(E)1411618).

##### Material examined.

(6 ♂ 7 ♀). New Caledonia • 1 ♂; Province Sud, Noumea, Parc Zoologique & Forestier; 22.2582°S, 166.458°E; alt. 84 m; 18 Sep. 2018; 22 Sep. 2018 em. [adult emergence]; Ohshima, Issei et al. leg.; Rearing No. IsO-1080; Host: “*Glochidion billarideri*” [recte billardierei]; DNA extraction No.: IO-853; RMNH.INS.1557476 • 2 ♂ 2 ♀; Province Sud, Province Sud, Noumea, Near Parc Zoologique & Forestier; 22.2548°S, 166.4527°E; alt. 83 m; 18 Sep. 2018; 20, 26–28 Sep. 2018 em.; Ohshima, Issei et al. leg.; Rearing No. IsO-1080; Host: “*Glochidion billarideri*” [recte billardierei]; DNA extraction No.: IO-851 & IO-967, IO-863, IO-854, IO-860; Genitalia slides: EvN5649 ♂, EvN5558 ♀, EvN5559 ♀; RMNH.INS.25649, RMNH.INS.25558, RMNH.INS.25559; RMNH.INS.1557475; RMNH • 1 ♂ 3 ♀; same collecting data as previous; 22, 26–28 Sep. 2018 em.; DNA extraction No.: IO-852, IO-855, IO-858, IO-862; KPU • 1 ♂ 1 ♀; same collecting data; 27 Sep. 2018 em.; DNA extraction No.: IO-856 (♂), IO-857 (♀); MNHN • 1 ♂ 1 ♀; same collecting data; 27–28 Sep. 2018 em.; DNA extraction No.: IO-859 (♀), IO-861 (♂); NHMUK.

##### Diagnosis.

**Figures 15–20.**
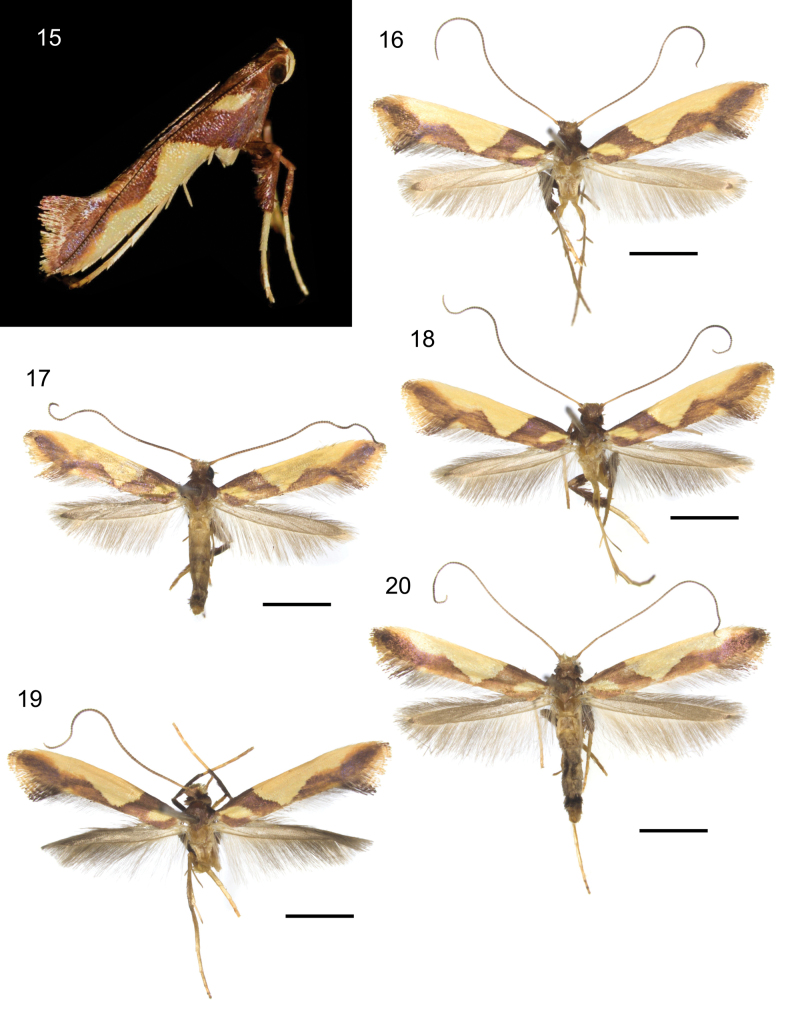
Habitus of adult *Caloptilia xanthopharella* (Meyrick, 1880). **15**. Resting posture, Australia, New South Wales, photograph Nick Lambert (https://www.inaturalist.org/observations/147192726); **16**. Female, RMNH.INS.25558; **17**. Male, RMNH.INS.1557475; **18**. Female, RMNH.INS.25559; **19**. Male, RMNH.INS.25649; **20**. Male, RMNH.INS.1557476. Scale bars: 2 mm.

*Caloptilia xanthopharella* adults closely resemble other species of *Caloptilia*. Like *C. deltanthes* (Meyrick, 1935) from French Polynesia, *C. xanthopharella* has a small blotch of pale yellow scales at the base of its forewings, but this blotch is triangular with one tip reaching the base and another almost reaching the dorsal edge in *C. deltanthes* while it is diamond-shaped and clearly separated from the wing edges in *C. xanthopharella* (Figs 15–20). *Caloptilia xanthopharella* can also be distinguished from other *Caloptilia* by its large pale-yellow forewing patch made of two merged triangles, the proximal with sharp edges and the distal with blurred edges. Males of this species can be distinguished from the other two New Caledonian congeners by its less dilated valva. Females can be easily recognised by its hammer-shaped signa. Larvae make a succession of a linear mine, a blotch mine, a tentiform mine and a leaf-roll typical for the genus *Caloptilia* on *Glochidion ferdinandi* (Figs 46, 47).

##### Description of adult.

***Habitus*** (Figs 15–20). Forewing length 4.95–5.69 mm (*n* = 5). No sexual dimorphism exhibited.

**Figures 21, 22.**
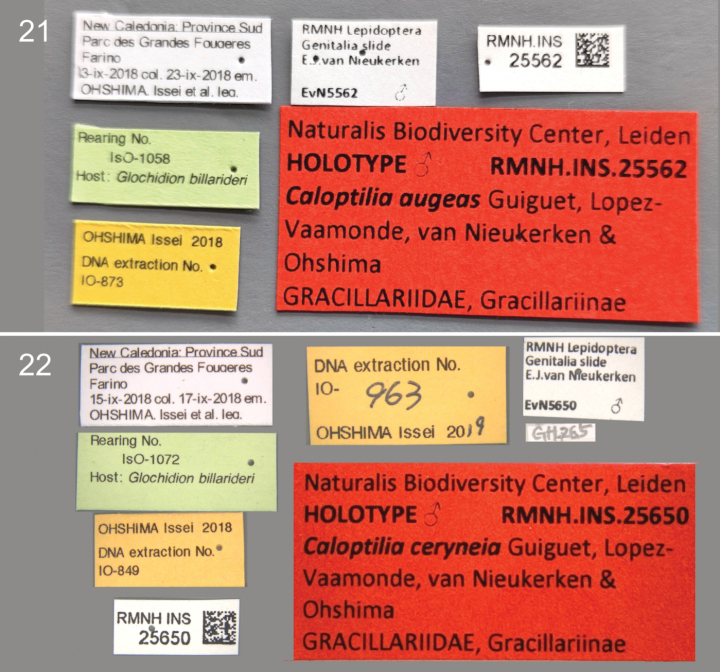
Labels of Holotypes. **21**. *Caloptilia augeas* holotype, male, RMNH.INS.25562; **22**. *Caloptilia ceryneia* holotype, male, RMNH.INS.25650.

***Head***: Vertex red-orange. Antenna 1.1 longer than forewing, brown, yellow near the base. Labial palpus upcurved, each segment pale yellow and brown at the tip.

**Figures 23–28.**
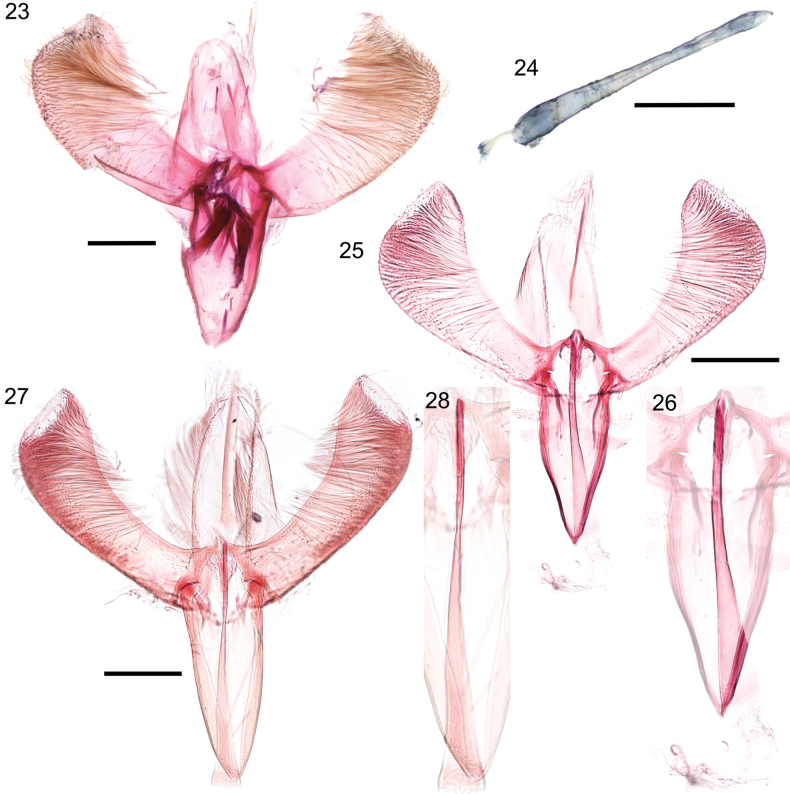
Male genitalia of *Caloptilia augeas* sp. nov., *C. ceryneia* sp. nov., and *C. xanthopharella* (Meyrick, 1880) in ventral view. **23**. *C. augeas* holotype, whole genitalia, with valvae spread out and phallus removed, genitalia slide EvN5562; **24**. *C. augeas* holotype, phallus; **25**. *C. ceryneia* holotype, whole genitalia, with valvae spread out and phallus in situ, genitalia slide EvN5650; **26**. *C. ceryneia* paratype, phallus; **27**. *C. xanthopharella*, whole genitalia, with valvae spread out and phallus in situ, genitalia slide EvN5649; **28**. *C. xanthopharella*, phallus. Scale bars: 0.2 mm.

***Thorax***: Tegula brown. Forewing rather broad, with ground of brown scales with metallic blue reflections; a small diamond-shaped blotch of pale-yellow scales at the base; first costal blotch triangular shaped with sharp edges from basal 1/4 to 1/2, pale yellow, reaching dorsum, connected with the second costal blotch, smaller than the first and with semi-circular and blurred edges, from beyond middle to the tip, pale yellow, not reaching dorsum; fringe brown shading to orange near the tip. Hindwing brown-grey; fringe brown-grey. Fore- and midlegs with coxa brown; tarsi pale yellow; hindleg with coxa pale yellow.

**Figures 29–31.**
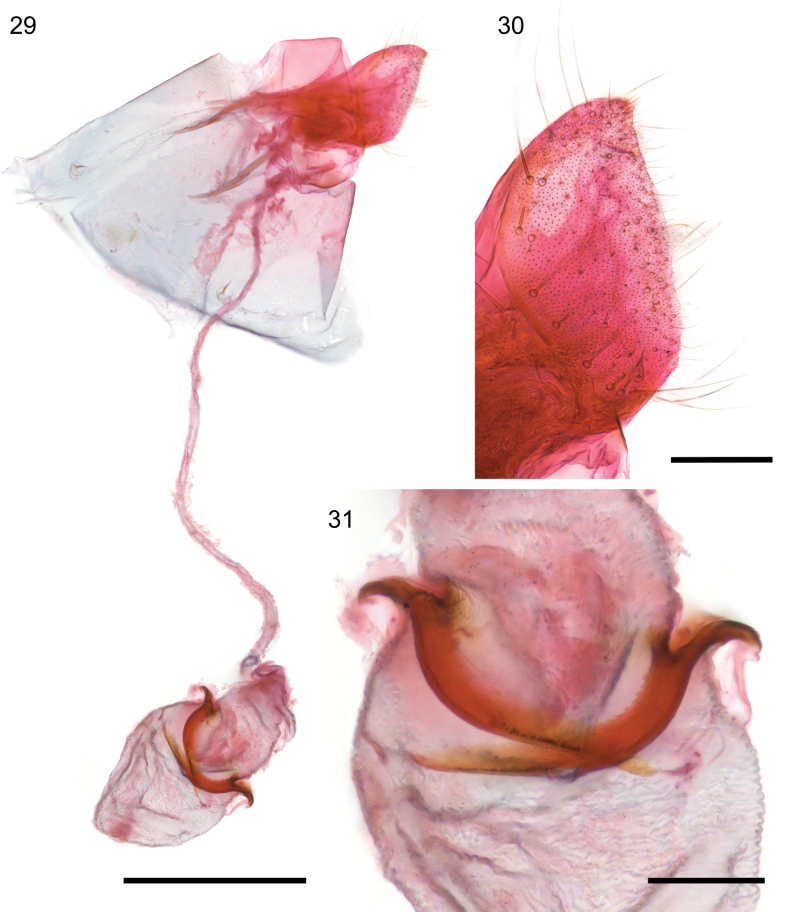
*Caloptilia augeas* sp. nov., female genitalia, paratype, genitalia slide EvN5561. **29**. Whole genitalia in dorso-lateral view; **30**. Papilla analis, lateral view; **31**. Top of bursa with signa. Scale bars: 0.5 mm (**29**), 0.1 mm (**30, 31**).

***Abdomen***: Terga pale yellow; sterna pale yellow.

***Male genitalia*** (Figs 27, 28): Valva spatulate, with the lower margin rounded; numerous long setae present along the apex and distal margin of the valva, covering the sacculus. Phallus needle-shaped, without cornuti, about as long as valva; phallus length 635 µm, valva length 570 µm.

**Figures 32–34.**
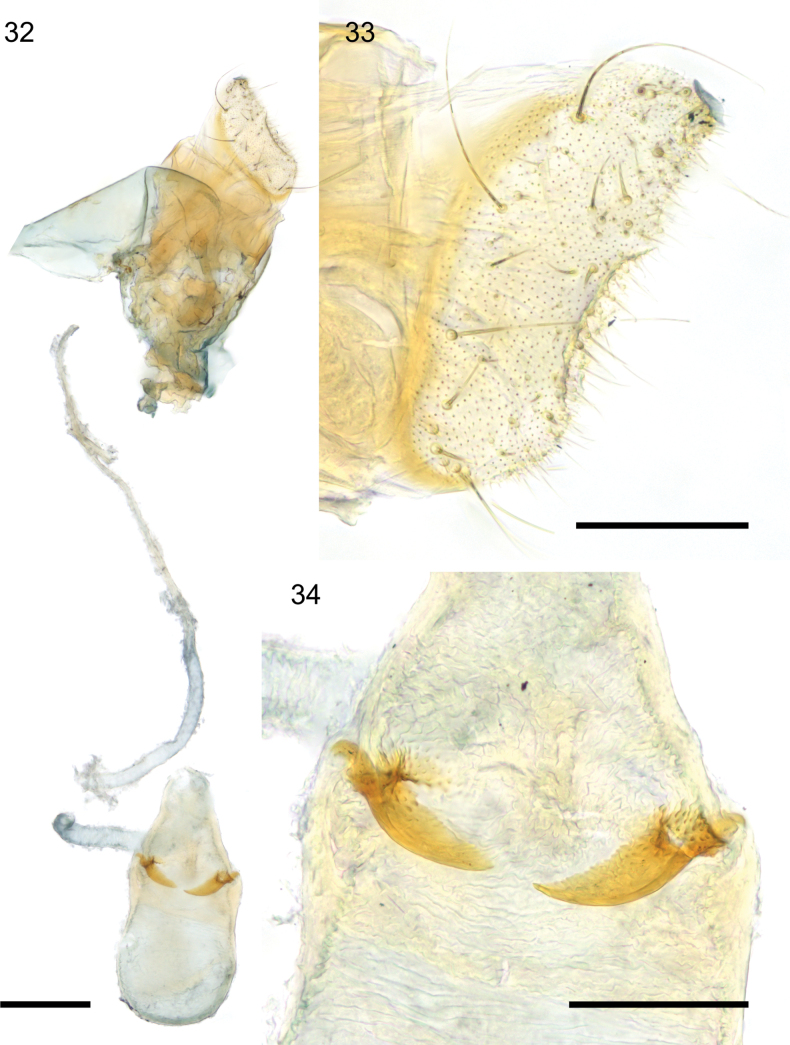
*Caloptilia ceryneia* sp. nov., female genitalia, paratype, genitalia slide EvN5565. **32**. Whole genitalia, ductus bursae broken; **33**. Papilla analis, lateral view; **34**. Top of bursa with signa. Scale bars: 0.2 mm (**32**), 0.1 mm (**33, 34**).

**Figures 35–37.**
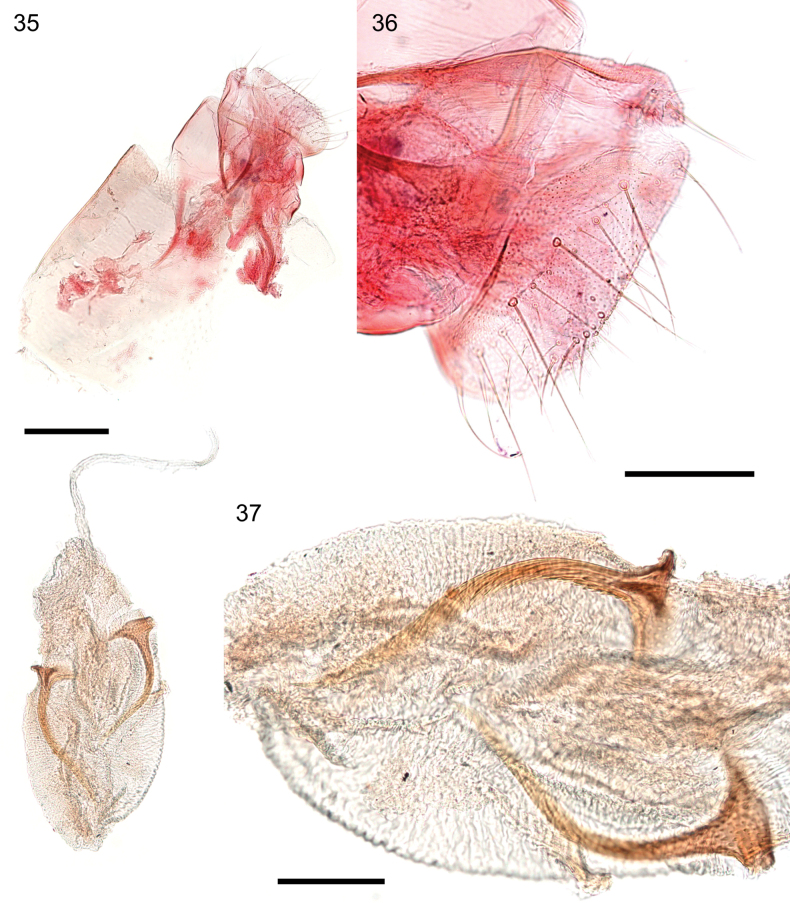
*Caloptilia xanthopharella* (Meyrick, 1880), female genitalia, genitalia slide EvN5559. **35**. Whole genitalia, ductus bursae broken; **36**. Papilla analis, lateral view; **37**. Top of bursa with signa. Scale bars: 0.2 mm (**35**), 0.1 mm (**36, 37**).

***Female genitalia*** (Figs 35–37): Papillae analis longer than wide, sparsely covered with long setae. Ductus bursae slender and membranous. Corpus bursae elongate-ovoid; signum consisting of two independent plates of similar size, each with a hammer-shaped head and a narrow, long, curved tail nearly as long as the diameter of the corpus bursae. Signa length 356–384 µm.

**Figures 38–42.**
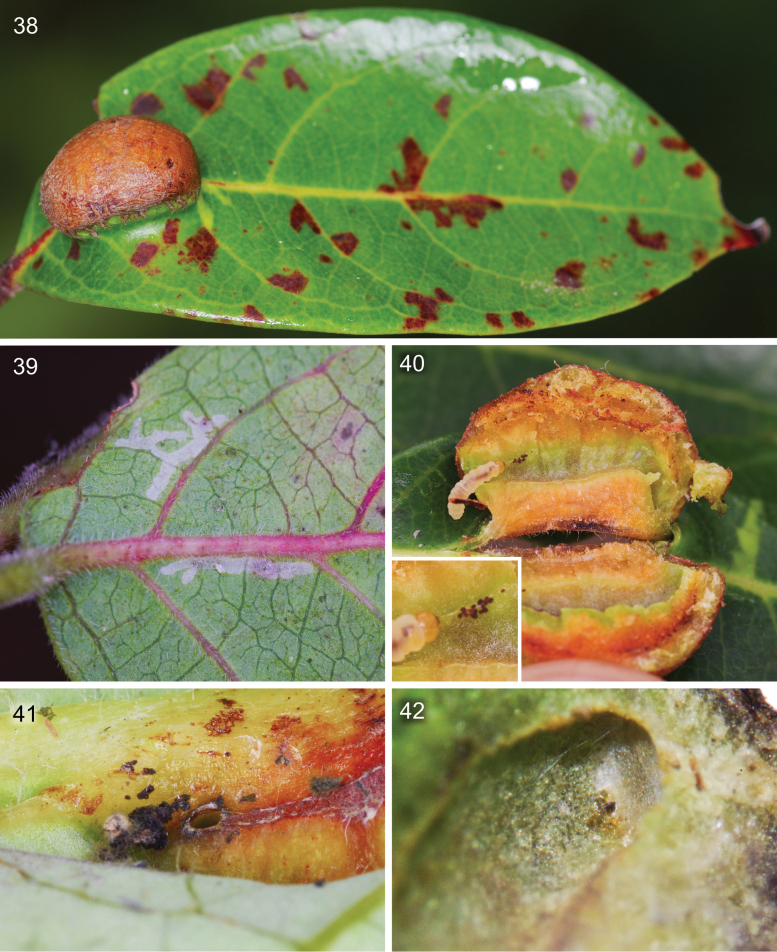
The gall of *Caloptilia augeas* sp. nov. on the leaves of *Glochidion billardierei* Baill. (Phyllanthaceae) collected in Parc des Grandes Fougères. **38**. The galls are located on the adaxial side of the leaf, usually near the midrib; **39**. Early instars make epidermal leaf-mines before inducing a gall; **40**. Gall cross section showing that the larval chamber is connected with the outside through a hole at the right extremity, detail showing the larva collecting frass with silk; **41**. External view of the frass excretion hole on the abaxial side of the galls; **42**. Internal view of the frass excretion hole showing the silk membrane closing the hole after use.

**Figures 43–45.**
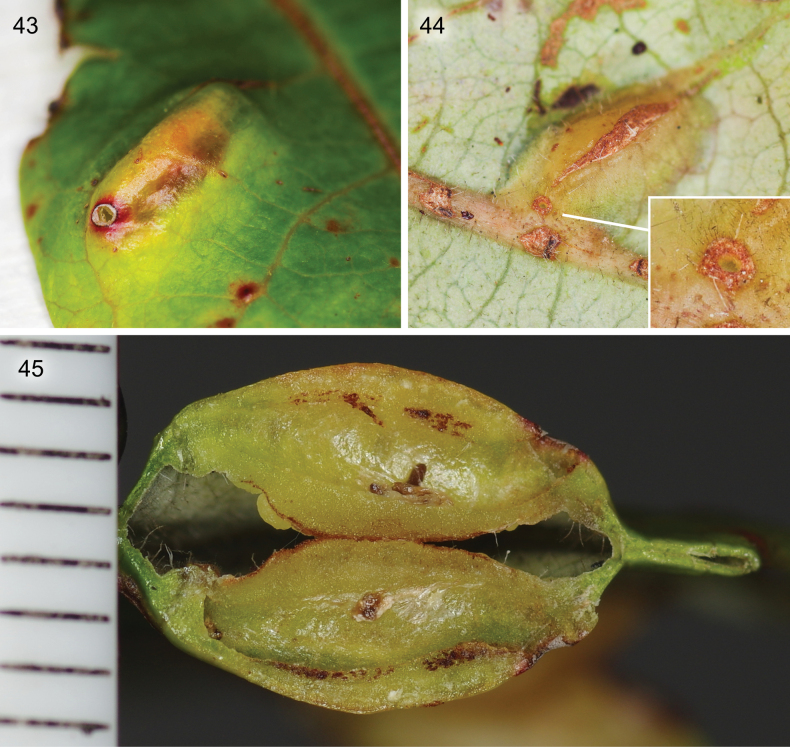
The gall of *Caloptilia ceryneia* sp. nov. on the leaves of *Glochidion billardierei* Baill. (Phyllanthaceae) collected in Parc des Grandes Fougères. **43**. Adaxial side view; **44**. Abaxial side view, detail showing the frass excretion hole; **45**. Cross section, with a millimetre scale on the left.

**Figures 46, 47.**
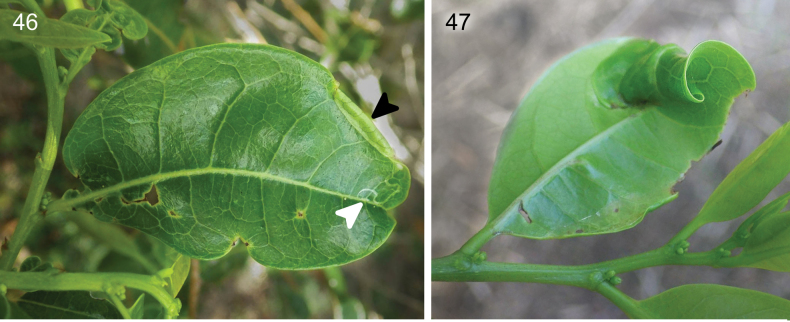
The mines and leaf-roll of *Caloptilia xanthopharella* (Meyrick, 1880) on the leaves of *Glochidion billardierei* Baill. collected in Noumea, near Parc Zoologique & Forestier. **46**. Epidermal linear mine (white arrow) and tentiform mine (black arrow) caused by early instars; **47**. Leaf-roll caused by late instars.

##### Biology.

In Australia, *C. xanthopharella* is known to feed on *Glochidion ferdinandi* (Müll. Arg.) F.M. Bailey (Common 1990). Here we present a new host plant record *Glochidion billardierei*. The larva first makes an epidermal linear mine on adaxial or abaxial surfaces, then makes a blotch mine it will turn into a tentiform mine (Fig. 46). The later instar larvae leave this tentiform mine and migrate to the tip of a neighbouring leaf to make a leaf roll (Fig. 4). After exiting the leaf roll, the larva prepares a cocoon on a neighbouring plant. Adults emerged between 22 and 26 September.

##### Distribution.

It is known to occur in Australia: coastal regions of New South Wales and Queensland, Fiji, Solomon Islands and Vanuatu (Meyrick 1880; Bradley 1957, 1962; De Prins and De Prins 2024). Here we record *C. xanthopharella* for the first time from New Caledonia.

##### DNA barcodes.

All 13 DNA barcodes obtained from New Caledonia cluster into a single haplotype, which groups together with 15 additional barcodes from across Australia within the same BIN, BOLD:AAI6656. This BIN shows an average distance of 0.96% and a maximum distance of 1.93%; there is a distance of 7.85% to an unnamed Australian *Caloptilia* with BOLD:AAW8621 (Suppl. materials 2, 3).

## Discussion

New Caledonia is renowned for its rich biodiversity and high level of local micro-endemism (Caesar et al. 2017). As with many other oceanic islands, its fauna and flora are highly disharmonic with certain groups being highly diversified (Platania et al. 2024). Within the Lepidoptera, for instance, the Micropterigidae have undergone a significant radiation in New Caledonia, resulting in at least 55 – as yet undescribed – species (Gibbs and Lees 2014). In contrast, the Gracillariidae, are poorly documented: only one introduced phyllocnistine species (*Phyllocnistis citrella* Stainton, 1856) and two genera, *Acrocercops* (Acrocercopinae) and *Epicephala* (Ornixolinae) have so far been recorded from the archipelago (Kawakita and Kato 2004; Gargominy et al. 2022). This is clearly an underestimate of the true diversity of New Caledonian Gracillariidae fauna, with many new species still awaiting formal description. Some of these, like the two *Caloptilia* species described here, are endemic, while others such as *C. xanthopharella*, may also be found in nearby regions such as Australia, particularly in the dry sclerophyllous forest.

Gall induction has evolved independently in at least eleven superfamilies within Lepidoptera: Nepticuloidea, Adeloidea, Gracillarioidea, Yponomeutoidea, Gelechioidea, Sesioidea, Choreutoidea, Carposinoidea, Tortricoidea, Alucitoidea and Pterophoroidea (Miller 2005). Within the family Gracillariidae, only four species are known to induce galls, each associated with a different plant organ. For example, *Epicephala obovatella* Kawakita & Kato, 2016 induces galls on the fruits of *Glochidion obovatum* Siebold & Zucc. (Kawakita and Kato 2016), *Kallia murtfeldtella* (Busck, 1904) on the stems of *Penstemon*, and both *Caloptilia cecidophora* and *Borboryctis euryae* Kumata & Kuroko, 1988 on the leaves of *Glochidion* (Kumata 1966; Guiguet et al. 2019) and *Eurya* (Guiguet et al. 2018), respectively. However, the number of known gall-inducing gracillariids is likely underestimated, as the two new leaf-galling species described here suggest.

Our DNA barcoding results support *C. augeas* sp. nov. and *C. ceryneia* sp. nov. as two distinct sister species. The larval behaviour we describe, where frass is excreted through a specialised opening in the gall, which is then temporarily sealed with silk is, to our knowledge, unique among gall-inducing insects. While some gall-inducing insects, such as aphids, maintain a permanent aperture for frass disposal (Wool 2005), this is the first documented case of temporary closure following frass expulsion.

Further research is needed to address several aspects of the biology of these two species. Specifically, it remains to be determined how many larval instars they undergo and whether, like *C. cecidophora*, they exhibit an additional sixth instar (Guiguet et al. 2019). It is also important to study their phenology, particularly whether they remain within the gall for extended periods, as in *Borboryctis euryae* (Guiguet et al. 2018). Moreover, the speciation process that leads to the divergence of *C. augeas* and *C. ceryneia* warrants closer investigation. Although these two species use the same host plant and occur sympatrically—galls of both species often coexist on the same tree, and occasionally even on the same leaf, they differ markedly in wing pattern. Yet, they exhibit only a shallow phylogenetic divergence (<4%) and no discernible morphological differences in male genitalia. Similar within-host speciation has been reported in gall midges of the genus *Asphondylia* (Joy and Crespi 2007), suggesting that the role of gall induction in facilitating such divergence should be explored in future studies. Our findings also highlight the need to investigate *Caloptilia* diversity across the broader Indo-Pacific region, particularly among species associated with Phyllanthaceae.

Finally, the discovery of these two new species highlights the ecological significance of Parc des Grandes Fougères, the type locality, as a key conservation area. The ongoing reforestation efforts within the park could benefit from strategic planting of *Glochidion* trees which may serve as pioneer species for habitat restoration. In turn, this would contribute to the conservation of the micromoth species described herein.

## Supplementary Material

XML Treatment for Caloptilia augeas

XML Treatment for Caloptilia ceryneia

XML Treatment for Caloptilia xanthopharella

## Additional information

### Use of AI

No use of AI was reported.

### Author ORCIDs

Antoine Guiguet https://orcid.org/0000-0002-3101-825X

Erik J. van Nieukerken https://orcid.org/0000-0002-5721-1840

David Giron https://orcid.org/0000-0001-8356-0983

Barbara Gravendeel https://orcid.org/0000-0002-6508-0895

Carlos Lopez-Vaamonde https://orcid.org/0000-0003-2278-2368

Issei Ohshima https://orcid.org/0000-0001-8295-9749

### Data availability

All specimen data and DNA data are available on Boldsystems and in GenBank (see https://doi.org/10.5883/DS-CALNEO). Relevant specimen data will be added to the Collection Registration System of Naturalis Biodiversity Center.

## Supplementary materials

Supplementary material 1 Specimen data of 59 specimens deposited in BOLD dataset DS-CALNEO (https://doi.org/10.5883/DS-CALNEO) and included in this study Data type xlsx https://binary.pensoft.net/file/1527632This dataset is made available under the Open Database License (http://opendatacommons.org/licenses/odbl/1.0/). The Open Database License (ODbL) is a license agreement intended to allow users to freely share, modify, and use this Dataset while maintaining this same freedom for others, provided that the original source and author(s) are credited. Antoine Guiguet, Erik J. van Nieukerken, David Giron, Barbara Gravendeel, Carlos Lopez-Vaamonde, Issei Ohshima

Supplementary material 2 Summary statistics for DNA barcodes of specimens included in this study Data type xlsx Explanation note N = number of specimens barcoded; Dmin_NN heterospecific = minimum distance to nearest neighbor; NN = nearest neighbor; BIN Dmean = average intraspecific distance within a BIN; BIN Dmax = maximum intraspecific distance within a BIN. https://binary.pensoft.net/file/1527633This dataset is made available under the Open Database License (http://opendatacommons.org/licenses/odbl/1.0/). The Open Database License (ODbL) is a license agreement intended to allow users to freely share, modify, and use this Dataset while maintaining this same freedom for others, provided that the original source and author(s) are credited. Antoine Guiguet, Erik J. van Nieukerken, David Giron, Barbara Gravendeel, Carlos Lopez-Vaamonde, Issei Ohshima

Supplementary material 3 Neighbor-Joining tree of 59 sequences and three BINs included in this study Data type pdf https://binary.pensoft.net/file/1527634This dataset is made available under the Open Database License (http://opendatacommons.org/licenses/odbl/1.0/). The Open Database License (ODbL) is a license agreement intended to allow users to freely share, modify, and use this Dataset while maintaining this same freedom for others, provided that the original source and author(s) are credited. Antoine Guiguet, Erik J. van Nieukerken, David Giron, Barbara Gravendeel, Carlos Lopez-Vaamonde, Issei Ohshima
