# Supplementary material for: Two new species of Caloptilia (Lepidoptera, Gracillariidae) from New Caledonia inducing galls on Glochidion billardierei (Phyllanthaceae) and redescription of C. xanthopharella (Meyrick, 1880)
Source: Zookeys. 2026 Feb 4;1268:113–37. doi: 10.3897/zookeys.1268.173885 (PMC12895181; doi:10.3897/zookeys.1268.173885)
Supplement: Supplementary material 3 — Neighbor-Joining tree of 59 sequences and three BINs included in this study [file zookeys-1268-113_article-173885__-s003.pdf]

BOLD TaxonID Tree

Title : Tree Result - DS-CALNEO (59 records selected)
Date : 30-May-2024
Data Type : Nucleotide
Distance Model : Kimura 2 Parameter
Marker : COI-5P
Colourization : [blue]=Stop Codons [red]=Contamination or misidentification

Label : Process ID
Label : Taxon
Label : Country
Label : Barcode Cluster (BIN)

Sequence Count : 59
Species count : 3
Genus count : 1
Family count : 1
Unidentified : 2

BIN Count : 3

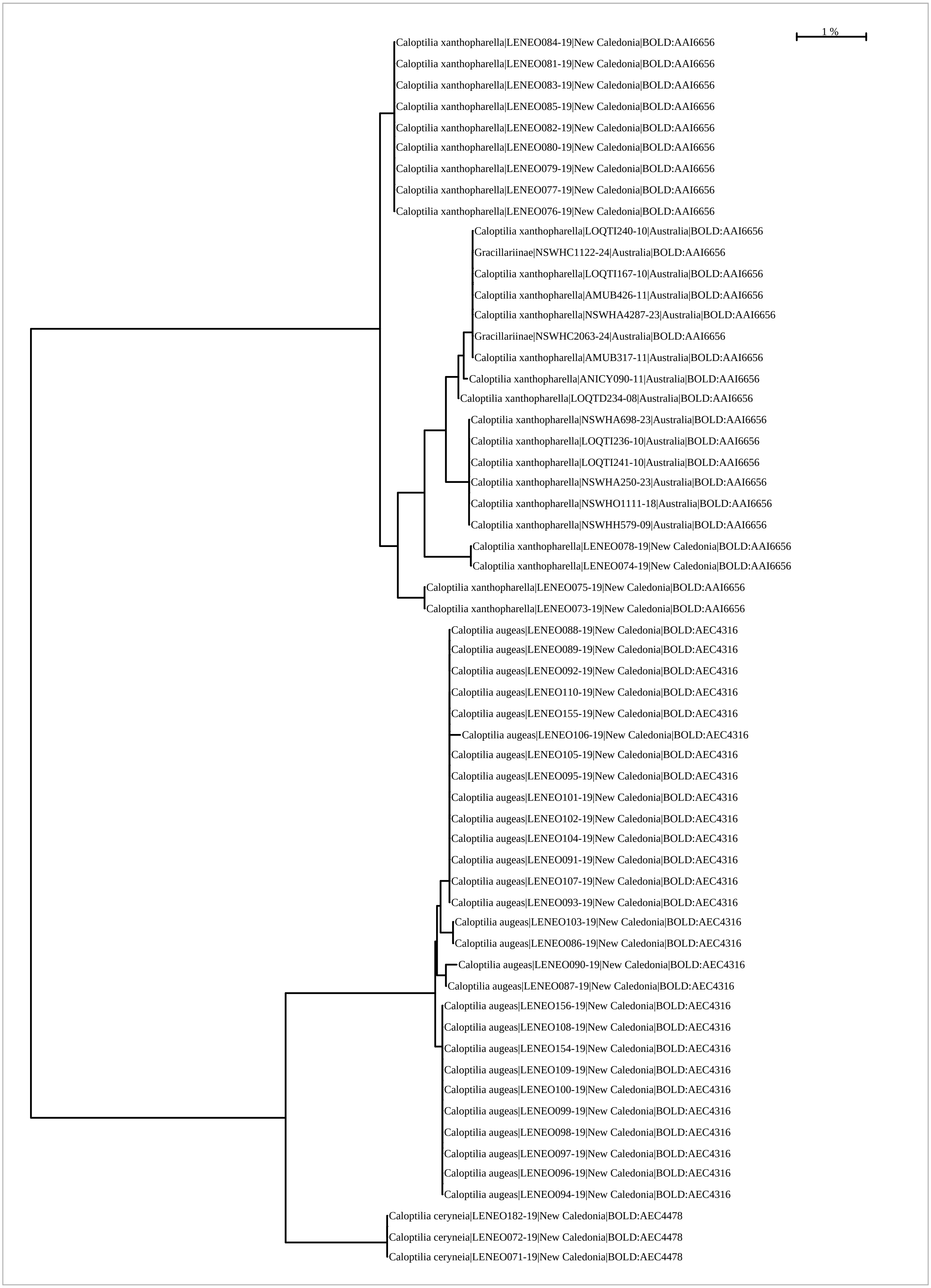
1 %
Caloptilia xanthopharella|LENEO084-19|New Caledonia|BOLD:AAI6656
Caloptilia xanthopharella|LENEO081-19|New Caledonia|BOLD:AAI6656
Caloptilia xanthopharella|LENEO083-19|New Caledonia|BOLD:AAI6656
Caloptilia xanthopharella|LENEO085-19|New Caledonia|BOLD:AAI6656
Caloptilia xanthopharella|LENEO082-19|New Caledonia|BOLD:AAI6656
Caloptilia xanthopharella|LENEO080-19|New Caledonia|BOLD:AAI6656
Caloptilia xanthopharella|LENEO079-19|New Caledonia|BOLD:AAI6656
Caloptilia xanthopharella|LENEO077-19|New Caledonia|BOLD:AAI6656
Caloptilia xanthopharella|LENEO076-19|New Caledonia|BOLD:AAI6656
Caloptilia xanthopharella|LOQTI240-10|Australia|BOLD:AAI6656
Gracillariinae|NSWHC1122-24|Australia|BOLD:AAI6656
Caloptilia xanthopharella|LOQTI167-10|Australia|BOLD:AAI6656
Caloptilia xanthopharella|AMUB426-11|Australia|BOLD:AAI6656
Caloptilia xanthopharella|NSWHA4287-23|Australia|BOLD:AAI6656
Gracillariinae|NSWHC2063-24|Australia|BOLD:AAI6656
Caloptilia xanthopharella|AMUB317-11|Australia|BOLD:AAI6656
Caloptilia xanthopharella|ANICY090-11|Australia|BOLD:AAI6656
Caloptilia xanthopharella|LOQTD234-08|Australia|BOLD:AAI6656
Caloptilia xanthopharella|NSWHA698-23|Australia|BOLD:AAI6656
Caloptilia xanthopharella|LOQTI236-10|Australia|BOLD:AAI6656
Caloptilia xanthopharella|LOQTI241-10|Australia|BOLD:AAI6656
Caloptilia xanthopharella|NSWHA250-23|Australia|BOLD:AAI6656
Caloptilia xanthopharella|NSWHO1111-18|Australia|BOLD:AAI6656
Caloptilia xanthopharella|NSWHH579-09|Australia|BOLD:AAI6656
Caloptilia xanthopharella|LENEO078-19|New Caledonia|BOLD:AAI6656
Caloptilia xanthopharella|LENEO074-19|New Caledonia|BOLD:AAI6656
Caloptilia xanthopharella|LENEO075-19|New Caledonia|BOLD:AAI6656
Caloptilia xanthopharella|LENEO073-19|New Caledonia|BOLD:AAI6656
Caloptilia augeas|LENEO088-19|New Caledonia|BOLD:AEC4316
Caloptilia augeas|LENEO089-19|New Caledonia|BOLD:AEC4316
Caloptilia augeas|LENEO092-19|New Caledonia|BOLD:AEC4316
Caloptilia augeas|LENEO110-19|New Caledonia|BOLD:AEC4316
Caloptilia augeas|LENEO155-19|New Caledonia|BOLD:AEC4316
Caloptilia augeas|LENEO106-19|New Caledonia|BOLD:AEC4316
Caloptilia augeas|LENEO105-19|New Caledonia|BOLD:AEC4316
Caloptilia augeas|LENEO095-19|New Caledonia|BOLD:AEC4316
Caloptilia augeas|LENEO101-19|New Caledonia|BOLD:AEC4316
Caloptilia augeas|LENEO102-19|New Caledonia|BOLD:AEC4316
Caloptilia augeas|LENEO104-19|New Caledonia|BOLD:AEC4316
Caloptilia augeas|LENEO091-19|New Caledonia|BOLD:AEC4316
Caloptilia augeas|LENEO107-19|New Caledonia|BOLD:AEC4316
Caloptilia augeas|LENEO093-19|New Caledonia|BOLD:AEC4316
Caloptilia augeas|LENEO103-19|New Caledonia|BOLD:AEC4316
Caloptilia augeas|LENEO086-19|New Caledonia|BOLD:AEC4316
Caloptilia augeas|LENEO090-19|New Caledonia|BOLD:AEC4316
Caloptilia augeas|LENEO087-19|New Caledonia|BOLD:AEC4316
Caloptilia augeas|LENEO156-19|New Caledonia|BOLD:AEC4316
Caloptilia augeas|LENEO108-19|New Caledonia|BOLD:AEC4316
Caloptilia augeas|LENEO154-19|New Caledonia|BOLD:AEC4316
Caloptilia augeas|LENEO109-19|New Caledonia|BOLD:AEC4316
Caloptilia augeas|LENEO100-19|New Caledonia|BOLD:AEC4316
Caloptilia augeas|LENEO099-19|New Caledonia|BOLD:AEC4316
Caloptilia augeas|LENEO098-19|New Caledonia|BOLD:AEC4316
Caloptilia augeas|LENEO097-19|New Caledonia|BOLD:AEC4316
Caloptilia augeas|LENEO096-19|New Caledonia|BOLD:AEC4316
Caloptilia augeas|LENEO094-19|New Caledonia|BOLD:AEC4316
Caloptilia ceryneia|LENEO182-19|New Caledonia|BOLD:AEC4478
Caloptilia ceryneia|LENEO072-19|New Caledonia|BOLD:AEC4478
Caloptilia ceryneia|LENEO071-19|New Caledonia|BOLD:AEC4478
